# Gaming well: links between videogames and flourishing mental health

**DOI:** 10.3389/fpsyg.2014.00260

**Published:** 2014-03-31

**Authors:** Christian M. Jones, Laura Scholes, Daniel Johnson, Mary Katsikitis, Michelle C. Carras

**Affiliations:** ^1^Faculty of Arts and Business, University of the Sunshine CoastMaroochydore, QLD, Australia; ^2^Science and Engineering Faculty, Queensland University of TechnologyBrisbane, QLD, Australia; ^3^School of Public Health, Johns Hopkins University, BaltimoreMD, USA

**Keywords:** videogames, mental health, well-being, PERMA, Seligman, flourishing

## Abstract

This paper is a review of the state of play of research linking videogaming and flourishing, and explores the role of videogames and technology to improve mental health and well-being. Its purpose is to develop understandings about the positive intersection of gaming and well-being, to document evidence regarding links between videogames and positive mental health, and to provide guidelines for use by other researchers as they design and use tools and games to improve mental health and well-being. Using Huppert's (Huppert and So, 2013) proposition that to flourish is more than the absence of mental disorder but rather a combination of feeling good and functioning effectively, resulting in high levels of mental well-being, and Seligman's (Seligman, 2011) PERMA theory of well-being, the paper identifies strengths in existing games that generate positive affect, positive functioning, and positive social functioning, contributing to, and supporting mental health and well-being.

## INTRODUCTION

Flourishing mental health has been defined as a combination of feeling good and functioning effectively resulting in high levels of mental well-being. To flourish is more than the absence of disorder with flourishing conceived as the opposite of mental disorder rather than its mere absence (Huppert and So, 2013). Mental disorders are universal and present in all people of all countries (World Health Organisation, 2001) and it is imperative that society gains a better understanding, enabling and encouraging flourishing to be achieved (Keyes, 2002; Seligman, 2011; Huppert and So, 2013). Responding to concerns about the well-being of young people and emerging evidence of positive impacts of videogames this paper explores the state of play of research linking videogame play and flourishing mental health. Young people have increasing access to computers and videogames with gaming environments moving from predominantly solitary states to multi player environments. “Videogames”, within the context of this paper, refers to electronic/digital games played on personal computers, home consoles (e.g., Microsoft Xbox, Sony Playstation, Nintendo Wii), tablets (e.g., iPads), mobile devices (e.g., smart phones, handhelds like Nintendo 3DS), and the web (e.g., via facebook or other websites). This review of the state of play of research linking videogaming and flourishing, and the role of videogames and technology, considers the potential of videogames to enhance mental health and well-being. While the paper focuses on the potential positive effects of videogaming, within the literature there has been a long history of highlighting the negative effects of videogames, and this contribution is duly noted.

## NEGATIVE EFFECTS OF VIDEOGAME PLAY

Traditionally, much of the research on videogames has focused on the negative effects of playing such games, and in particular the effects of playing violent videogames. This research has provided insights into the ways that pre-existing characteristics may lead to some young people being vulnerable to negative impacts of videogames although further research is needed. Increasingly, the impact of violent videogames is being considered from a more nuanced perspective with an understanding that publication bias and the emphasis on the use of laboratory measures of aggression may exaggerate relationships between videogame violence and aggression, and not accurately predict real life behavior (Sherry, 2004, 2007; Ferguson, 2007; Kutner and Olson, 2008; Boyle et al., 2011). Various studies focus on longitudinal measures that attempt to demonstrate causal relationships between violent videogames and aggression. Many of these studies rely on self-reported measures of aggressive feelings or attitudes (Shibuya et al., 2008; Möller and Krahé, 2009; Anderson et al., 2010; Lemmens et al., 2011), while other studies include self-reported counts of aggressive behaviors (Shibuya et al., 2008; Bucolo, 2011), or combined teacher and peer ratings (Gentile and Gentile, 2008). While some of these studies do report associations between earlier violent videogame play and later self-reported aggression (Möller and Krahé, 2009; Anderson et al., 2010; Bucolo, 2011), or combined peer-and teacher-reported aggression (Gentile and Gentile, 2008), others do not support long-term direct effects of violent videogames on self-reported physical aggression (Shibuya et al., 2008; Lemmens et al., 2011). Interestingly, violence has been found not to be an important factor in contributing to game enjoyment as players play violent games for the same reasons they play other games, such as enjoyment of the challenge and the freedom to act in a virtual world (Przybylski et al., 2009a).

In a similar manner there have been studies concerned with pathological gaming. Longitudinal research on pathological gaming, however, is relatively scarce. One study examined the psychosocial causes and consequences of pathological gaming among adolescents, finding lower psychosocial well-being was generally an antecedent of pathological gaming (Lemmens et al., 2011). Diminished social competence, increased loneliness, and lower self-esteem were linked to an increase in pathological gaming six months later (Lemmens et al., 2011). Findings suggest that lower psychosocial well-being was more likely to be a cause rather than a consequence of pathological gaming (Chak and Leung, 2004; Ko et al., 2005). Studies on small groups of players who spend excessive amounts of time on games have shown that symptoms of addiction can arise including withdrawal, preoccupation, loss of control, and interpersonal or intrapersonal conflicts (Grüsser et al., 2007; Gentile, 2009), however, other studies fail to support links between heavy play and negative psychosocial outcomes in non-addicted gamers (Lemmens et al., 2011; Van Rooij et al., 2011).

While the negative effects of playing videogames are well documented, many of the potential problematics of gameplay appear associated with excessive amounts of time immersed in play and links to existing lower psychosocial well-being. These findings suggest consideration of the negative and positive effects of videogame play is warranted. This said, there remains a gap in literature that explores the potential positive outcomes of moderate videogame play including the many creative, social, and emotional benefits from playing videogames, including violent games (Ryan et al., 2006; Kutner and Olson, 2008; Wang et al., 2008; Przybylski et al., 2009a; Allahverdipour et al., 2010). Considering the potential of videogames for enhancing positive well-being is the focus of the following sections.

## VIDEOGAMES FOR POSITIVE WELL-BEING

Recently, there has been significant interest in the links between videogame play and positive well-being (see Durkin and Barber, 2002; Barr et al., 2006; Colwell, 2007; Ryan and Deci, 2008; Wang et al., 2008; Hull, 2009; Allahverdipour et al., 2010; Boyle et al., 2011; Przybylski et al., 2011; Snodgrass et al., 2011b). There is also increased concern that the potential value of videogames has not been sufficiently considered particularly in terms of the benefits for young people at risk (Kutner and Olson, 2008). Existing literature on gaming has been inconsistent and has often focused on aggression. However, over the last five to ten years, increasing attention has been given to the possibility of games improving health and well-being (Desai et al., 2010). A number of studies reflect this shift and consider a nuanced approach to the positive and negative influences of game play with a number of significant studies demonstrating clear benefits to individuals who spend time in game play. As contemporary research provides examples of the benefits of gaming, the question becomes more about optimal levels of game play, the influence of factors such as gender, subgroups, and associated experiences, and the interplay of particular genres on well-being.

Studies focusing on the potential positive outcomes of videogame play have found links to positive emotions for players (Ryan et al., 2006; Kutner and Olson, 2008; Wang et al., 2008; Przybylski et al., 2009a; Allahverdipour et al., 2010). Moderate videogame play has been found to contribute to emotional stability (Przybylski et al., 2011) and reducing emotional disturbances in children (Hull, 2009). Significantly, videogame play has been advocated as a means of relaxation and stress reduction by regular players (Russoniello et al., 2009; Wack and Tantleff-Dunn, 2009; Snodgrass et al., 2011b). Children playing games have reported “letting off steam” in response to problems with friends or parents with feelings of anger, guilt, or frustration dissipating after time spent in game play resulting in players feeling much happier (Colwell, 2007). Interestingly, depressed mood has been found to be significantly lower in moderate players of videogames compared to those who “never” play videogames and those who play videogames to excess (Durkin and Barber, 2002), and time spent in gameplay is highlighted as the moderating factor. Videogame play has also been linked to the development of skill acquisition (Gee, 2008; Johnson et al., 2011), and has been found to contribute to individual attitude and behavior changes (Baranowski et al., 2008; Burns et al., 2010). While videogames have been shown to provide a range of positive outcomes the focus of this paper is on links between videogaming and flourishing mental health as this association has previously not been clearly articulated.

## FLOURISHING MENTAL HEALTH

Flourishing adults demonstrate high levels of emotional well-being, are happy and satisfied, tend to see their lives as having a purpose, feel some degree of mastery and accept all parts of themselves (Keyes, 2002). Furthermore, they have a sense of personal growth and have a sense of autonomy and an internal locus of control, choosing their fate in life instead of being victims of fate (Keyes, 2002). To explore the potential of videogame play in enhancing well-being this review considers positive research findings within Seligman’s positive psychology model for well-being (Seligman, 2011). Seligman’s model of well-being contains five elements: Positive emotion; Engagement; Relationships; Meaning and purpose; and Accomplishment (PERMA). Using Seligman’s PERMA positive psychology model we have reviewed research linking videogames and flourishing mental well-being.

### POSITIVE EMOTION

The ability, opportunity, and experience of feeling positive emotions such as happiness, satisfaction, joy, and the many other descriptors of good feelings.

### ENGAGEMENT

The opportunity to be fully engaged and completely immersed in activities. To be engaged is to be so immersed in an activity that you lose your sense of time and feel boundless energy.

### RELATIONSHIPS

The nature of a person’s relationships strongly correlate to their happiness, health, and overall well-being. Improving relationships can bring greater happiness.

### MEANING

Having meaningful activities brings a sense of purpose and fulfillment to daily life. A sense of purpose often accompanies activities which contribute to something larger than self.

### ACCOMPLISHMENT

Having goals and objectives to accomplish brings a sense of achievement and satisfaction to life, contributing to the feeling of well-being. This is the sense of satisfaction experienced when checking off tasks from a daily to-do list.

The awareness of PERMA can help increase well-being by focusing on combinations of feeling good, living meaningfully, establishing supportive and friendly relationships, accomplishing goals, and being fully engaged with life. We argue that videogames are an environment for nurturing these experiences that can help players go beyond surviving to really flourishing in life.

## VIDEOGAMES, PERMA AND WELL-BEING

We propose that videogames, by their very nature, have design elements aligned with attributes of well-being, and that playing videogames can provide opportunities for flourishing mental health. This paper fills a gap in literature by explicitly reviewing emerging research to examine the positive outcomes of videogaming (Kutner and Olson, 2008; Allahverdipour et al., 2010). This review is timely as videogame use is widespread. Over 95% of homes with children under the age of 18 have a device for playing videogames, and 94% of children aged between 6 and 15, and 90% of people aged 16 to 25 play videogames (Brand, 2012). In 2005 the average age of a gamer was 24, in 2011 this average age rose to 32 (Brand, 2012). Videogames are no longer a solitary activity with 70% of players playing videogames with others either in the same room or over the internet (Brand, 2012). There have also been changes in terms of female gaming and in the 7 years from 2005 to 2011, the proportion of gamers who were female increased steadily from 38 to 47% with equal representation of female gamers to males predicted to be imminent (Brand, 2012). Therefore any mental health gains experienced by gamers could be increasing, and widespread. With the significance of the potential benefits of positive gameplay foremost, the following section details emerging research within the PERMA model.

## POSITIVE EMOTION

Emerging research suggests that moderate game play can contribute to positive emotions (Ryan et al., 2006; Kutner and Olson, 2008; Wang et al., 2008; Przybylski et al., 2009a; Allahverdipour et al., 2010) and emotional stability (Przybylski et al., 2011). Positive mental well-being has also been associated with game play as a means of relaxation and stress reduction (Russoniello et al., 2009; Wack and Tantleff-Dunn, 2009; Snodgrass et al., 2011b). The amount of game play, however, is a moderating factor on the player’s personal well-being. Durkin and Barber’s (2002) examination of the relationship between game play and several measures of adjustment for 1304 high school students, found that videogame play was unlikely to be harmful and instead was often associated with positive outcomes. There were advantages for those adolescents who occasionally played videogames (low use) and those who played daily (high use) compared to the young people who reported that they never played games (never; Durkin and Barber, 2002). Specifically, depressed mood was significantly lower in the “low” use group compared to the “never” and “high” groups who reported similar outcomes. Self-esteem was also higher in the “low” use group with self-concept regarded higher by players than non players, with “high” use players scoring the highest in this domain. Both groups of players also reported higher levels of family closeness and less risky friendship networks than non-players, with attachment to school also higher in these two videogame play groups.

Allahverdipour et al. (2010) similarly found that middle-school students with moderate amounts of gameplay reported better mental health compared to non-gamers and excessive gamers. Gamers spent an average of 6.3 h per week playing video games with 47% reporting that they had played one or more intensely violent games including: Dead or Alive, Def Jam, Doom, Driver, Mortal Kombat, Grand Theft Auto, Resident Evil, and Prince of Persia. Moreover, 92% of boys and 96% of girls played video games although boys typically played games for greater duration than girls. However, it is the amount of gameplay that appears significant with moderate gaming among young men providing a healthy source of socialization, relaxation, and coping (Wack and Tantleff-Dunn, 2009). Defining “non-gamers” as those who did not play at all, “low” 1–6 h per week, “moderate” 7–10 h per week, and “excessive” as more than 10 h per week, the study found a curvilinear relationship between videogame playing and mental health outcomes with “moderate” gamers faring best (Allahverdipour et al., 2010). Although “excessive” gamers showed mild increases in problematic behaviors (such as somatic symptoms, anxiety, and insomnia, social dysfunction, and general mental health status), it was non-gamers who indicated the poorest outcomes on these constructs. Non-gaming has been found to put boys, in particular, at greater risk for problems. This effect for non-gamers has also been reported by others who found gaming positively contributed to creative, social, and emotional benefits (Kutner and Olson, 2008).

There is concern that the potential benefits of videogames (including some games with violent content) have not received enough attention. Kutner and Olson (2008), co-directors of the Harvard Medical School Center for Mental Health and Media, recently found that boys who did not play any videogames during a typical week had a high risk of emotional disturbance. Boys have been found to use videogames for emotional regulation, to help them relax, to forget problems, or to feel less lonely (Kutner and Olson, 2008). While the survey, that formed part of this study, did find correlations between Mature-rated violent gameplay and some common childhood problems, such as aggressive behaviors or school problems, this risk was for both boys and girls. However, results did not show causality and most children who played violent games did not have problems. In fact many of the boys describe using violent videogames to manage their emotions and to deal with anger, frustration, and stress (Keyes, 2002; Kutner and Olson, 2008).

Videogames have also been reported to be used for stress relief. Game play for children has been used as a means of mood alteration or “letting off steam,” following problems at school, or with friends, or with parents (Colwell, 2007). Furthermore, children have reported an understanding of the mood altering benefits of their videogame play and explicitly make choices to engage with videogames to manage their emotions. While videogame play has been associated with positive emotion, it has also been linked to immersive states of engagement.

## ENGAGEMENT

Engagement within a task has been associated with increased happiness (Killingsworth and Gilbert, 2010). Engagement refers to an emotional involvement or commitment to some object or domain of interest and to the experiential intensity of a relationship or interaction. It also refers to one’s temporal involvement or interactions with activities and social partners in the immediate environments with a strong relationship demonstrated between engagement and positive well-being (Shernoff, 2012). Intrinsically, interesting activities that evoke intense concentration and enjoyment have been described as creating flow or optimal engagement and experience (Csikszentmihalyi, 1998, 2008). Pointedly, videogame players commonly report optimal experiences reaching deeply “immersive” states of consciousness, in some cases growing to feel like they actually are their characters and really in the game (Snodgrass et al., 2011b). Csikszentmihalyi considered that flow would require many years of learning and skill development and that many would not experience flow. However, videogames through physical input, adjustment of difficulty, and real-time visual feedback, can provide near immediate and on demand flow experiences for their players (McGonigal, 2011).

This engagement with gaming has seen 9.6 million players of World of Warcraft (WoW), a massively multiplayer online (MMO) game (Stickney, 2013), collectively spend over six million years in the game. With the primary task within the game to improve oneself, players get a sense of blissful productivity (Bardzell et al., 2008). Players level up through completing quests to make their in-game avatar stronger, quicker, and better equipped. With these new skills and stronger character, players are able to take on more difficult quests and subsequent further leveling up. Leveling up can take in excess of 500 h, and for many gamers this isn’t the endgame but instead only the start of their engagement with WoW.

Part of the allure of WoW has been associated with the immersive experiences players report. Snodgrass et al. (2011a, b, 2012) examined different types of videogaming experiences and the effects they can have on players’ lives, including their levels of stress, satisfaction, and happiness. Central was the premise that WoW and similar games can be thought of as new “technologies of absorption” and contemporary practices that can induce dissociative states in which players attribute dimensions of self and experience to in-game characters, with potential psychological benefit or harm (Snodgrass et al., 2011a). The Snodgrass study found that half of respondents reported WoW as increasing their happiness, that WoW helped them “relax and combat stress,” and increased their “life satisfaction” (Snodgrass et al., 2011b). Two-thirds of their WoW respondents reported having at times immersive experiences.

Findings affirm that dissociation is positive and normal and that altered “absorbed” states of consciousness that many gamers reached (such as the “dissociative” identification some gamers have with their characters), provide some gamers with relaxation as well as some of the most satisfying, meaningful experiences of their lives (Snodgrass et al., 2011b, 2012). Experiences of “absorption-dissociation” explain the positive therapeutics of the game, which combines relaxation alternating with mildly stress-inducing flow states (Snodgrass et al., 2011b). Positive stress helps players achieve “flow” and the experience of being in the “zone,” as players are pushed by the game’s tasks and challenges where there is likelihood of experiencing success. The two states of experience and consciousness are achieved if players imaginatively immerse themselves in this game-world and strongly identify with their character-avatars. This deep immersion can divert attention from real-world stress, allowing gamers to more readily reach deeply relaxed, even meditative, states of play (Snodgrass et al., 2011b). While the positive benefits of playing WoW have been described, the amount of game play can become problematic with time becoming a significant factor and influential on potential well-being outcomes. Again, the amount of gameplay has been found to be significant in moderating the potential well-being benefits (Durkin and Barber, 2002; Allahverdipour et al., 2010), making time spent in play a significant consideration for enhancing well-being.

## RELATIONSHIPS

While immersive states have been associated with engagement in videogames, videogaming environments also provide opportunities to develop and maintain positive relationships. Positive relationships are considered highly important for the psychosocial adjustment and well-being of children, adolescents and adults (Bagwell and Schmidt, 2011). How positive relationships are defined is transitioning, as many young people form and maintain what they consider friendships online (Amichai-Hamburger et al., 2013). MMO gamers under the age of 18 have reported that the friendships they form online are comparable or better than their real-life friendships (Yee, 2006). WoW players report creating social capital through online gameplay with players using the game to extend real life relationships, meet new people and form relationships of varying strengths (Williams et al., 2006).

It has been argued that on-line communication is being used to enhance both the quantity and quality of communication between friends, leading to greater closeness and intimacy (Valkenburg and Peter, 2011). Playing online with friends who are also friends in real life can be healthy as interactions have helped regulate game play (Snodgrass et al., 2011a). Playing with real life friends has allowed players to transfer positive gaming experiences into real life. However, playing with real friends makes it harder to immerse, impacting on some of the stress reduction benefits although also potentially reducing the risk of problematic play and addiction. Playing with real life friends has also allowed players of WoW to share their experiences of success and achievement, to bolster and repair their feelings of worth and esteem as players. Players are then able to transfer in-game accomplishments and status to their real-life networks of friends and family. Playing WoW in this way creates cognitive and social bridges between online and offline worlds providing more objective perspective on MMO use and allowing better self-regulation. Therefore, playing with friends has the potential to affect levels of problematic play by mediating immersion and enhancing real-life relationships increasing social and psychological resilience (Colwell, 2007; Hull, 2009; Wack and Tantleff-Dunn, 2009; Snodgrass et al., 2011a; Trepte et al., 2012). The social interactions that occur within and outside of MMO play have been found to be highly social providing opportunities to create strong friendships and emotional relationships with a high percentage of gamers making life-long friends and partners (Yee, 2006; Cole and Griffiths, 2007).

Massively multiplayer onlines’ are online “places” in which social interaction can occur and are unique in the fact that they collect and mix people pursuing goals in three-dimensional space (Williams et al., 2006). WoW is a vibrant social space for millions of players, populated with a range of social experiences ranging from ephemeral impersonal groups to sustained and deep relationships that extend offline. Games such as WoW include structure and rule sets impacting on what kinds of people play, what they do, and how and why they interact with one another. As part of gameplay social organizations are created with the design encouraging the formation of persistent player associations (Taylor, 2003).

Genres of videogames other than MMOs also offer opportunities for socializing with other players. For example first-person-shooters and sport games almost always include online multiplayer modes that allow for both competitive and cooperative play. Similar modes are also sometimes included in action games, platforming games, puzzle games, and other genres. To this end, Johnson and Gardner (2010) found differing genres of games to result in varying experiences of autonomy but similar levels of competence and relatedness.

Positive relationships within online videogames have been associated with opportunities for social and emotional support. In a recent study, two-fifths of study participants said they would discuss sensitive issues with their online gaming friends that they would not discuss with their real life friends, with female players more likely to do this (Cole and Griffiths, 2007). Two-fifths of participants had met their online friends in real-life, suggesting that online videogaming is a social activity that facilitates social connections. A third of participants were attracted to another player (26% males compared to 42% females) suggesting that MMOs offer a safe environment for players to become emotionally involved with others. It would appear that videogames allow players to express themselves in ways they may not feel comfortable doing in real life because of their appearance, gender, sexuality, and/or age.

Online social videogames such as  (Words with Friends (WwF, 2013; similar to Scrabble) are encouraging families and friends to keep in touch. These games alert players to their turn and players can chat and/or leave messages for other players. In this manner, the game can be an excuse and mechanism for conversations to occur, with mothers and daughters discussing every day events and exchanging messages of affection. Playing these asynchronous games, where both players do not need to be online at the same time and can play their turn when convenient can encourage players to continue to exchange regularly (McGonigal, 2011). This “stickiness” (the ability of the game to keep the players playing) draws together the players to build and maintain positive relationships.

## MEANING

Involvement in meaningful activities brings a sense of purpose and fulfillment to daily life. Finding purpose has been associated with involvement in activities which contribute to something larger than self. McGonigal (2011) reports that videogame players have a sense that they are part of something bigger than themselves. She details that in the final campaign in Halo 3, when gamers must protect the Earth from alien attack, players collective completed over 10 billion kills (achieved April 2009), at 12,000 kills a minute. Although there is no real value in killing an alien in Halo 3, McGonigal (2011) suggests that this does not mean they do not have meaning. Meaning is significant not just to ourselves, friends and family, but rather to a much larger group such as the whole Human race. Seligman (1998) suggests that the larger the group you attach yourself to, the more meaning you can derive. Connecting with millions of videogame players across the world, against a common in-game attack, is bigger than any one player and this has been associated with deriving meaning and subsequently well-being. As testament to the popularity of connecting with other players, in eight years of Halo 3 play, players compiled more than 123 billion hours of gaming, or more than 85 million days, achieving more than 136 billion kills and earning over 79 billion medals (in-game awards based on a player’s actions during a typical multiplayer match) for their gaming.

Another example of players working together with purpose is evident when WoW players work together in guilds. They often participate in highly structured organizational experiences working towards common goals. Guilds can have relaxed social structures, known as “the tree house,” or adopt highly structured, hierarchical organization known as “the barracks” (Williams et al., 2006). Players in formally structured guilds tend to have a more social experience than others. Playing WoW is thus like a team sport, which has its own rules, literal boundaries, and social norms (Williams et al., 2006). There are however, self-initiated tactics, team strategies, styles, and goals that make the play space a stage for socialization, organization, and networks. Some guilds rely on relatively haphazard policies and procedures, and are more likely to contain social tensions, misunderstandings, and fights. Guilds with clear policies and procedures manage tasks better and have generally happier members (John et al., 1991).

Videogames also have the potential to help eliminate loneliness. Even when friends and family are far away, videogames can allow players to interact, share, and be social (Pwn or Die Blog, 2009). Meeting online to play together in a game can provide strong social and emotional ties, and more meaning for society both within and beyond the game.

## ACCOMPLISHMENT

Working towards goals and objectives to accomplish can contribute to a sense of achievement and satisfaction in life. This in turn contributes to feelings of competence and well-being. Ryan et al. (2006) suggested that the psychological “pull” of games is largely due to their capacity to engender feelings such as competence therefore enhancing psychological wellness. Competence is a psychological need for challenge and feelings of effectance. Therefore, factors that enhance the experience of competence (such as opportunities to acquire new skills or abilities, to be optimally challenged, or to receive positive feedback) enhance perceived competence. Competence is enhanced in gaming contexts where game controls are intuitive and readily mastered, and tasks within the game provide ongoing optimal challenges and opportunities for positive feedback (Ryan et al., 2006). Ryan et al. (2006) found the desire for future play was predicted by feelings of presence and players’ self- determined needs for competence, autonomy, and relatedness.

In a similar manner to competence, accomplishment can also be linked to videogame play. To this end, Yee (2006) and Suznjevic and Matijasevic (2010) found accomplishment was an important motive for playing MMOs. Female players were more driven by the relationships and more likely to use the MMO environment to build supportive social networks, confirming findings reported by Cole and Griffiths (2007) and Snodgrass et al. (2011a), that online gaming can support positive relationships. However, male players were significantly more likely to be driven by accomplishment (Yee, 2006). The “achievement” factor measured the desire to become powerful in the context of the virtual environment through the achievement of goals and accumulation of items associated with power. While some users participated in the environment to make friends and form supportive social networks, others used the environment to become powerful through the accomplishment of goals.

Structured goal setting (i.e., incorporating factors of goal quality, Clark et al., 2009) has been shown to stimulate motivation and promote goal attainment (Locke, 1996). Goal attainment in turn is associated with significant benefits to health and well-being. Studies have found that goal progress enhances positive affect, improves life satisfaction and enhances general well-being (Carver and Scheier, 1990; Sheldon and Kasser, 1995; Ryan and Deci, 2000). This suggests that not only does goal progress enable a person to attain the target goal; it also produces general benefits to overall well-being. When structured goal setting is applied within tertiary mentoring programs students will not only improve their academic performance but will also experience enhanced general well-being.

Whereas accomplishments can be infrequent, unrecognized, and sometime unachievable in real-life, within the videogame accomplishments are regular, achievable and immediately rewarding. Videogames often have clear tasks to complete (e.g., quests), instructions to complete the task, and the task can be challenging but achievable, aligning with the player’s current skill and experience. The player also receives immediate rewards and recognition when the task is completed (McGonigal, 2011). Players have a specific goal, an understanding of how to achieve the goal, the ability to complete the goal and importantly, the motivation to want to take on the goal. As (Seligman (2004, p.40) argues “The most important resource building human trait is productivity at work.” Game players can be highly productive in the games they play, accomplishing and achieving, and improving skills that contribute to positive experiences and enhanced self-worth.

## CONCLUSION

As videogame play increases, we are moving towards a billion videogamers in the world. This review of the literature would suggest that videogame play has the potential to enhance life satisfaction and improve individual player’s mental well-being. The inherent nature of videogames appears to promote and facilitate all aspects of Seligman’s PERMA model, e g., Positive Emotion, Engagement, Relationships, Meaning and Accomplishment. Considering game play within this model suggests that play can have a positive effect on the mental health of individuals in society engaged in moderate videogaming.

Positive emotion contributes to the make-up of happiness and well-being. Literature provides links between positive mental well-being and play, providing illustrations of where game play provides a means of relaxation and stress reduction (Russoniello et al., 2009; Wack and Tantleff-Dunn, 2009; Snodgrass et al., 2011b). Although the amount of game play has been highlighted as a moderating factor, studies have shown that not playing videogames can result in the poorest well-being outcomes.

Engagement takes place when an experience becomes so absorbing that individuals completely lose sense of time. Achieving this state of flow or total engagement can be a natural outcome of being involved in activities they individuals love. Gaming activities that embody immense concentration and enjoyment have been described as creating flow or optimal experience (Csikszentmihalyi, 1998, 2008). Videogame players often report optimal experiences reaching deeply “immersive” states of consciousness (Snodgrass et al., 2011b) and leading to a sense of flourishing.

Relationships play a significant role in contributing to happiness and psychological health. Social relationships with strangers as well as with peers, siblings, parents, extended family, and friends are all sources of positive emotions and support. Online communication can enhance both the quantity and quality of communication between friends, leading to greater closeness and intimacy (Valkenburg and Peter, 2011) and young games have reported that the friendships they form online are comparable or better than their real-life friendships (Yee, 2006). In-game relationships offer social and emotion support, where players are able to discuss sensitive issues and exchange messages of affection.

Meaning, or belonging to, and serving something bigger than one’s self, contributes to flourishing well-being. Happiness comes from creating and having meaning by attaching to something larger than oneself such as an interactive gaming environment. This experience instils a sense that there is a larger purpose to life, and that individuals are part of this meaning. Having connections with something bigger can also be an effective barrier against depression, with the larger the group to which you attach the more meaning you can derive (Seligman, 1998). Connecting with players across the world in a multiplayer videogame is bigger than any one player, fostering meaning and well-being.

Accomplishment includes having explicit goals in life, and achieving these is important to well-being and happiness. Achievement helps to build self-esteem and can provide a sense of accomplishment, strengthening self-belief. Games have a psychological “pull” largely due to their capacity to engender feelings such as competence and competence forms part of gameplay when game controls are intuitive and readily mastered with the task providing optimal challenges and opportunities for positive feedback. Whereas accomplishments can be infrequent, unrecognized, and sometime unachievable in real-life, within the videogame accomplishments are regular, achievable, and immediately rewarding.

This review presents literature to evidence how moderate levels of videogame play can have a positive influence on well-being. Specifically, videogame play has been found to lead to improved mood, reduced emotional disturbance, improve emotion regulation, relaxation, and stress reduction. Importantly, moderate play has been associated with better outcomes than either excessive play or a lack of play. There is a lack of negative impact for the majority of young players, and instead videogame play is associated with greater self-esteem regarding intelligence, computer skills, and mechanical ability. The experience of feelings of competence, autonomy and relatedness during videogame play has been linked with higher self-esteem and positive affect.

The research reviewed suggests that the experience of engagement while playing videogames can have a positive influence on well-being. However, it is excessive game play that can become problematic. This is supported by studies in this review that articulate how the amount of game play has been found to be significant in moderating the potential well-being benefits (see Durkin and Barber, 2002; Allahverdipour et al., 2010). This finding, however, should be considered in tandem with findings identifying the influence of the nature of engagement (harmonious or obsessive, Przybylski et al., 2009b) as well as the influence of pre-existing psychosocial vulnerability (Lemmens et al., 2011; Van Rooij et al., 2011).

Videogame research must move beyond a “good–bad” dichotomy and develop a more nuanced understanding about videogame play. Future research should attempt to identify the causal relationships, including bidirectional effects, between time spent on games, and positive, as well as negative outcomes. Furthermore, an exploration of whether time spent in game is associated with better outcomes such as depressed mood and loneliness is warranted. Evaluation of moderators such as gender and pre-existing psychosocial well-being are also vital to incorporate. Research is also needed to explore the possibility that games can be designed to moderate in-game time such that players achieve optimal well-being and flourishing.

## Conflict of Interest Statement

The authors declare that the research was conducted in the absence of any commercial or financial relationships that could be construed as a potential conflict of interest.
